# Changes in Water Dynamics by Osmolytes Regulate Enzyme Activity

**DOI:** 10.1021/acs.jpcb.5c07605

**Published:** 2026-01-06

**Authors:** Sachika Furukawa, Mafumi Hishida

**Affiliations:** Department of Chemistry, Faculty of Science, Tokyo University of Science, 1-3 Kagurazaka, Shinjuku, Tokyo 162-8601, Japan

## Abstract

Although water is considered to play a crucial role in biological functions, the relationship between the water’s molecular dynamics and enzyme activity has not been systematically clarified. In the present study, we investigated the effects of osmolytes on enzyme activity by focusing on the dynamics of the surrounding water. Degradation of amylose by α-amylase (iodine–starch reaction) was monitored by visible light absorption to determine reaction rates. Terahertz time-domain spectroscopy was used to probe the collective rotational dynamics of water molecules on a picosecond time scale, allowing us to assess changes in water dynamics caused by osmolytes. We identified a clear correlation between enzyme activity and water dynamics beyond the molecular species of osmolytes: osmolytes that enhanced water mobility, such as urea, accelerated enzymatic reactions, whereas those that restricted water mobility, including sugars and polyols, suppressed enzymatic reactions. This correlation was consistently observed not only for osmolyte–water binary solutions but also in enzyme–osmolyte–water ternary systems, strongly implying that osmolytes affect enzyme activity indirectly through modifications of the surrounding water dynamics. These findings provide experimental evidence that enzymatic reactions are highly sensitive to picosecond-scale solvent dynamics. Osmolytes probably function as modulators of enzyme activity through their hydration effects. By highlighting the central role of water dynamics in enzymatic catalysis, this study deepens our understanding of the molecular mechanisms underlying enzyme function and offers a framework for interpreting osmolyte effects in biological systems.

## Graphical abstract

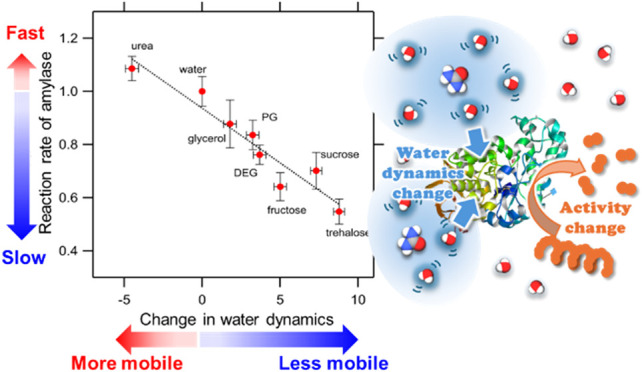

## Introduction

“What is the role of water in biological phenomena?” is one of the crucial issues in biological and material sciences.^1–6^ Importance of water in biological phenomena has been identified in phenomena such as thermal stability of proteins,^7–10^ ligand binding,^11–14^ enzyme activity,^15,16^ and lipid bilayers.^17–20^ For example, the structuring of the hydrogen bond network in water significantly affects the free energies of folded and unfolded protein,^7–10^ and a protein is stabilized by the excitation of hydration water.^21^ In enzymatic processes, not only the water structures but also the hydration water dynamics are considered important for the cooperation of protein dynamics with rearranging hydrogen bonds.^4,15,22^ Although the importance of the microscopic structure and water dynamics in individual phenomena is clear, a systematic understanding of these phenomena has not yet been achieved. Furthermore, although hydration is believed to extend over several layers of water molecules,^10,17,20,23^ how the state of hydration water over such long distances is related to the functions of biological molecules is not fully understood.

Regarding the role of water in determining the properties of proteins, the effects of small organic molecules called osmolytes, such as urea and sugars, have attracted wide attention. Osmolytes strongly affect structural stabilization and inhibit or promote protein denaturation.^10,24–27^ Although these stabilizing/destabilizing effects have been attributed to interactions between osmolyte molecules and proteins,^28–30^ several phenomena have been reported that cannot be explained by this mechanism.^31–33^ Recently, another mechanism has been suggested, as osmolytes contribute to the stabilization or destabilization of the folded structure of proteins by altering the hydrogen bonding and/or molecular dynamics of water around the protein.^24,27,34,35^ Recently, we clearly showed that the extent of inhibition or acceleration of water mobility by 15 osmolyte molecules strongly correlates with their effects on the denaturation temperature of ribonuclease A using terahertz time-domain spectroscopy (THz-TDS), which can analyze long-range hydration, including weakly affected hydration water.^27^ Kossowska et al. also confirmed that osmolyte molecules change the protein folded structure by altering the hydrogen bond structure of water around and inside myoglobin.^36^ The indirect effect of osmolytes through water on protein stability has become widely accepted in recent years.^15,24,35,37^ This indirect effect via water is similar to the mechanism of the salting-out of proteins, which depends on the ion species (Hofmeister effect).^38,39^ A similar mechanism for osmolytes was confirmed not only for proteins but also for synthetic polymers.^40^

In contrast to the effects of osmolytes on protein stability, their effects on enzyme activity, one of the most important protein functions, have not been well investigated. The effect of osmolytes on enzymes has only been studied in terms of the stability and preservation of enzyme activity,^41–46^ as the maintenance of enzyme activity is likely related to the stability of the folded structure. For example, betaine and trimethylamine N-oxide (TMAO) enhance the thermal stability of enzymes and preserve their activity at low concentrations.^41^ Sucrose and glycine protect enzymes from denaturation, stabilize their active conformations, and reduce their inactivation rates.^42^ They counteract the effects of denaturants, preserving enzyme activity and structural integrity.^43^ Glycerol and betaine maintain the maximum reaction rate and Michaelis constant in crowded environments, with minimal perturbation of enzyme kinetics.^44^ Trehalose and proline maintain the activity of PersiXyn2 at high levels for several days compared to those without the addition of an osmolyte.^45^ Wlodarczy et al. reported that several osmolytes are effective in improving the activity and stability of the anticancer enzyme l-asparaginase II; in particular, sucrose and sorbitol increased the specific activity of the enzyme by up to 70% and reduced aggregation.^46^ However, the effect of osmolytes on enzyme activity (i.e., the reaction rate) has barely been studied. In addition, the effect of changes in the water state caused by osmolytes on enzyme activity has not been studied.

Although the dynamics and hydrogen bond structure of water near the enzyme surface are considered important for enzyme functions, many aspects remain unclear. For instance, the mobility of water at the active site can influence the formation of enzyme–substrate complexes, thereby affecting reaction rates.^22^ The stretching vibrations of water have been reported to couple with the catalytic activity of pepsin.^47^ Increased water mobility, particularly in the presence of salts, has been correlated with enhanced enzyme activity, indicating that water facilitates the conformational changes necessary for catalysis.^48^ If differences in the state of water affect the reaction rates of enzymes, osmolytes should also indirectly affect enzymatic reactions through changes in the state of water.

In this study, we demonstrated the effect of osmolytes on the activity of a model enzyme, α-amylase, focusing on the state of water in the enzyme/osmolyte solutions. As we studied protein stability,^27^ we analyzed the effects of various osmolytes on water in a unified manner and found a strong correlation between α-amylase’s reaction rate and the mobility of surrounding water molecules. This strong correlation provides strong evidence that osmolytes indirectly contribute to the enzyme reaction rates by changing the state of water. The change in water mobility caused by osmolytes was observed using THz-TDS, which enabled us to detect changes in the collective reorientation dynamics of water on the picosecond time scale.^10,20,27,49–51^ THz-TDS can quantify the amount of hydration water—not only water molecules strongly hydrated by the solute, but also those weakly hydrated by the solute.^20,50^ As such a long-range hydration has been deemed important in protein stabilization,^10^ THz-TDS is one of the most appropriate techniques for clarifying the role of water in enzyme activity.

## Materials and Methods

### Materials

α-Amylase from bacillus amyloliquefaciens, 0.1 mol/L phosphate buffer solution (pH 6.4), trehalose dihydrate (≥98.0%), sucrose (≤100%), propylene glycol (PG, ≥99.0%), glycerol (≥99.5%), and diethylene glycol (DEG, ≥99.0%) were purchased from FUJIFILM Wako Pure Chemical (Osaka, Japan). Potato-derived amylose, iodine solution (Lugol’s solution: 3.4 g/L I_2_ and 6.8 g/L KI), and urea (≥98.0%) were obtained from Sigma-Aldrich (St. Louis, MO, U.S.A.). D-(−)-Fructose (≥98.0%) was procured from Nacalai Tesque (Kyoto, Japan). All the chosen osmolytes were neutrally charged, which prevented changes in pH and electrostatic interactions with amylase.

### Sample Preparation

To obtain the complex dielectric function using THz-TDS and investigate the hydration effects of osmolyte molecules, each osmolyte was dissolved in ultrapure water (Milli-Q, 18.2 MΩ·cm) at a concentration of 0.75 mol/L. For spectral analysis (details are described in the Results and Discussion section), the density of each solution was measured at room temperature (23.0 ± 1.0 °C) using DMA 35 (Anton Paar GmbH). THz-TDS measurements were performed on the osmolyte/water and the amylase/osmolyte/water systems. Solutions were prepared by dissolving amylase in a 0.75 M osmolyte solution so that the amylase concentration was 10 wt%.

To investigate the effects of osmolytes on the rate of amylose degradation by α-amylase, separate amylose/iodine/osmolyte and amylase solutions were prepared. For the amylose solution, 15 mg of amylose was dissolved in about 100 g of ultrapure water (Milli-Q) at about 90 °C. Two days later, the amylose solutions were passed through a filter with pores 200 nm in diameter to remove undissolved residue; therefore, the amylose concentrations of the samples varied. Then, 300 μL of iodine–potassium iodide solution (Lugol’s solution) was added to 100 g of the amylose solution. Each osmolyte was dissolved in the solution to a concentration of 1 mol/L. Separately from the amylose/iodine/osmolyte solutions, 1wt % of α-amylase was dissolved in 0.1 mol/L phosphate buffer as an enzyme solution. For visible light absorption spectroscopy, 1 mL of the α-amylase solution was added to 3 mL of the amylose/iodine/osmolyte solution, that is, the osmolyte concentration was 0.75 mol/L.

### Terahertz Time-Domain Spectroscopy

The THz-TDS measurements were performed using homemade equipment. The experimental setup is described in the literature.^27^ An infrared (IR) ultrafast pulse fiber laser (FemtoFErb780, TOPTICA (Munich, Germany); 780 nm, <100 fs, 100 MHz) was used as the light source. The IR light was bifurcated using a beam splitter to generate and detect THz waves. The IR light for THz detection passed through a delay stage to change its optical path length. THz wave emission and detection were performed using dipole photoconductive antennas (SD-TX101, SD-RX101, Pioneer (Tokyo, Japan)). The generated THz wave was focused to some extent by a superhemispherical silicon lens and then focused onto the sample position using a plastic lens. Lock-in detection was used for precise detection of the THz waveform in the time domain. An attenuated total reflection (ATR) setup with silicon dove prisms (refractive index = 3.4) was applied to the sample cell to accurately measure the complex dielectric functions of aqueous solutions.^52,53^ The THz waves were *p*-polarized at the prism surface such that even small changes in the dielectric constant induced by the solute hydration effect could be detected. The penetration depth of the evanescent field is 20 μm. The temperature of the ATR sample cell was controlled at 20.0 ± 0.1 °C using a Peltier device with PID control (TDC-1010A, Cell System (Osaka, Japan)). The entire system, including the fiber laser, was purged with dry air (QD-20–50 and RD-45-N, IAC (Kanagawa, Japan)). The reliable frequency range of the measured complex dielectric function is 0.3–2.5 THz.

### Visible Light Absorption Spectroscopy

To determine the reaction rate constant of amylose degradation by α-amylase, the time variation in visible light absorbance by the amylose/iodine/osmolyte solution after the addition of amylase was measured using an ASV11D spectrophotometer (AS ONE (Osaka, Japan)). For the time-variation measurements, we used a UV-3150PC spectrophotometer (SHIMADZU (Kyoto, Japan)) to determine the absorption wavelength of the amylose/iodine solution (Figure S1). The intensity of this peak at 615 nm decreased with amylose degradation. The enzyme solution (1 mL) was added to 3 mL of the amylose/iodine/osmolyte solution in a 1 cm × 1 cm quartz cell, and the absorbance at 615 nm was measured every 5 s for 10 min while stirring the solution. The absorbance from 15 s after the start of the measurement was analyzed to avoid large operating errors during stirring. Ultrapure water (4 mL) was used to calibrate the absorbance. All measurements were performed at room temperature (20.0 ± 0.5 °C). To confirm reproducibility, the reaction rate constants in urea and trehalose solutions were measured 13 and 12 times, respectively. The other osmolyte solutions were measured nine times each (Figure S2).

## Results and Discussion

To evaluate the reaction rate of amylase in osmolyte solutions, we measured the iodo-starch reaction rate. In the iodine-starch reaction, iodine penetrates into the helix structure of amylose, causing the solution to turn blue-violet (absorbance at ∼610 nm).^54^ α-Amylase is comprehensively known to hydrolyze glycosidic bonds in a two-step process in the Asp–Glu–Asp active site.^55,56^ In the process, enzyme-sugar intermediate state is formed at first, followed by hydrolytic cleavage of the glycosidic bond. The cleavage of the glycosidic bonds of amylose induces the breaking of the helix structure and fade of the blue-violet color. That is, progress of the reaction is evaluated by change in the visible-light absorbance. After the addition of the amylase solution to the amylose/iodine/osmolyte solution, the change in absorbance at 615 nm owing to amylose degradation was measured. The osmolyte concentration during the degradation reaction was 0.75 mol/L. The typical time dependence of absorbance is depicted in Figure 1. Because amylose degradation can be assumed to be a first-order reaction,^57–59^ the time dependence was fitted to a single exponential function (eq 1) to determine the reaction rate constant, *k*, for each osmolyte solution.

1 $$\mathrm{Abs}.=B+A_{0}e^{-\mathrm{kt}}$$

where *t* is the elapsed time, *B* is the background absorption of the respective solution, and *A*_0_ + *B* is the initial absorbance ( at 15 s after the start of the reaction).

**Figure 1:**
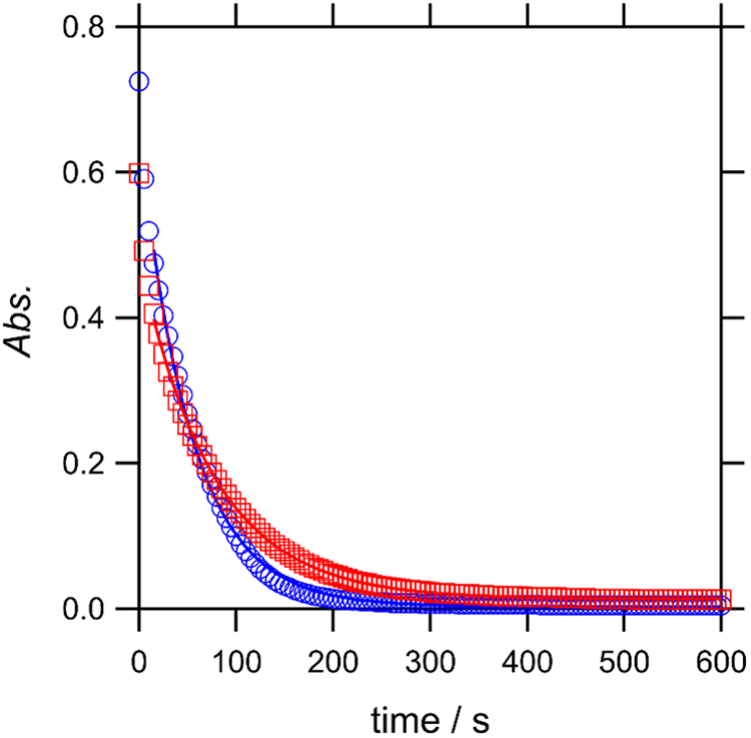
Change in absorbance at 615 nm for the amylose/iodine/osmolyte solutions after the addition of amylase. The blue circles and red squares represent amylose/iodine (without osmolyte) and amylose/iodine/fructose solutions, respectively. The solid lines represent the results of fitting with a single exponential function (eq 1).

All experiments were conducted 2 days after preparing the amylose solutions, as *k* decreased with increasing number of days after preparation (Figure S3). The measured *k* values of amylase in the amylose/iodine/urea solution are shown in Figure 2. The bars filled with the same colors in Figure 2(a) indicate the *k* value in the urea solution when five different amylose solutions were used. The value of *k* was found to strongly depend on the amylose solution and was reproducible only when the same bottle of amylose solution was used. This was probably due to the varying concentrations of amylose depending on the sample bottles owing to filtration during sample preparation. To eliminate the effect of varying amylose concentrations between different sample bottles, the *k* value for each osmolyte solution was normalized to the reaction rate in pure water (*k*_water_), for which the same bottle of amylose solution was used (shaded bar in Figure 2(a) in each color). Independent of the amylose solution bottle, the normalized reaction rate, *k*/*k*_water_, was reproduced well, as shown in Figure 2(b). Five amylose solutions containing urea were prepared, and 13 *k*/*k*_water_ results were well reproduced, as shown in Figure 2(b). Thus, *k*/*k*_water_ accurately represents the effect of osmolytes on the amylase activity. The *k*/*k*_water_ values for each osmolyte solution, determined by averaging multiple results, are shown in Figure 2(c) and Table S1 along with their standard deviations. The standard deviations of multiple results in the respective osmolyte solutions were relatively small, confirming that *k*/*k*_water_ reflects the effect of each osmolyte on the amylase reaction rate with good reproducibility. The normalized *k*/*k*_water_ depends on the amount of osmolyte added.

**Figure 2:**
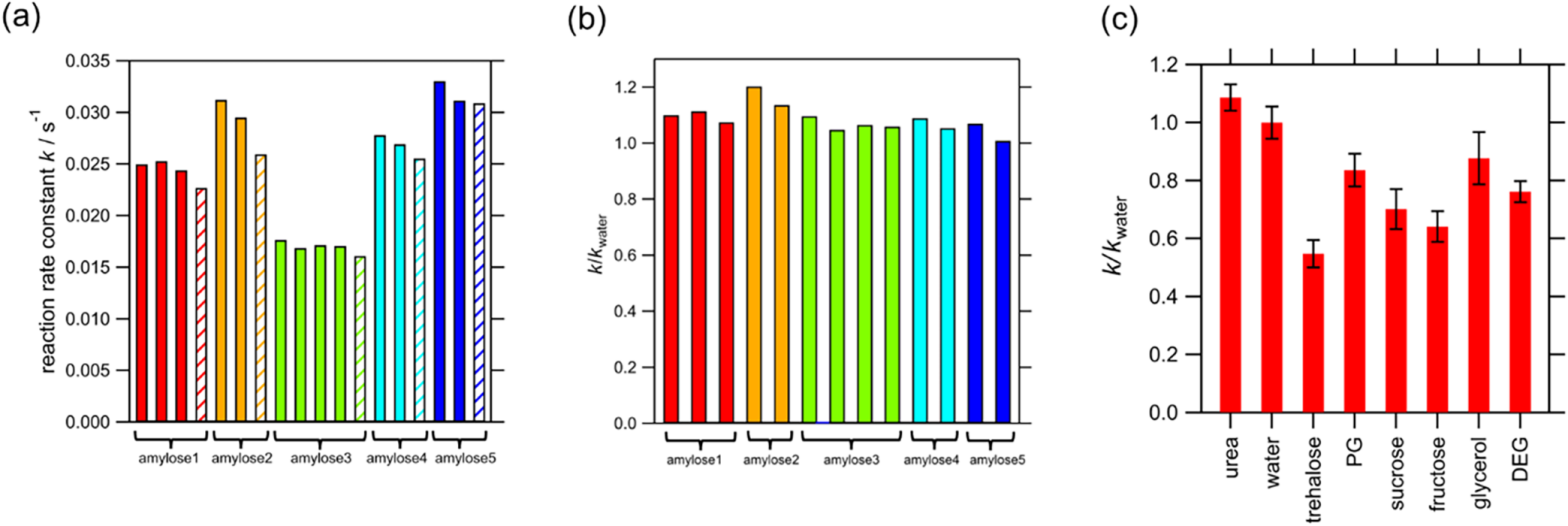
Reaction rate constant, *k*, of amylase in amylose/iodine/osmolyte solution. (a) *k* in amylose solutions with urea (filled bars). Identically colored bars indicate the results after using the same amylose solution. Because the concentrations of amylose varied among the sample bottles, the results were normalized by the reaction rate constant in the same amylose solutions without osmolyte (*k*_water_, shaded bars). (b) Normalized reaction rate constant (*k*/*k*_water_) for each amylose solution with urea. (c) Mean normalized reaction rate constant (*k*/*k*_water_) for amylase in amylose solution with each osmolyte. Error bars are the standard deviation after multiple measurement (e.g., 13 results for the solution with urea, (b)).

Figure 3(a,b) show the imaginary part of the dielectric constant, ε”, of pure water, 0.75 mol/L osmolyte solutions and amylase/osmolyte solutions measured using THz-TDS. The signal in this frequency range originates mainly from “slow relaxation” owing to the collective reorientation dynamics of water (relaxation time τ_slow_ ∼ 6 ps), “fast relaxation” owing to the dynamics of isolated water molecules from the hydrogen bond network (relaxation time τ_fast_ ∼ 0.3 ps), and intermolecular stretching vibrations (frequency, ω_s_ ∼ 6 THz).^51,60^ The hydration state of the solute was evaluated using a method described in the literature.^10,17,19,20,27,53,61,62^ As the concentrations of the osmolytes were low in this study, and the osmolytes were expected to absorb much fewer terahertz waves than water, the absorption of THz waves by the osmolytes can be ignored in the following analysis. In the present study, the dynamic modes of the hydration water and bulk water were assumed to be separated, as in the literature.^10,27,62^ The relaxation time of bulk water was not affected by the solute, whereas that of hydration water was variable. The hydration effect of the solute should significantly influence the slow relaxation mode of water. When water molecules are bound by the solute, the collective dynamics of water become slower, and the slow relaxation mode shifts to much lower frequencies than the THz range, as has been reported in many samples.^63–66^ In this case, the measured slow relaxation modes in THz region originated only from bulk-like water. Because some water molecules become hydrated and are not observed in the THz region, the intensity of the slow relaxation from bulk-like water decreases depending on the amount of hydration water, resulting in a decrease in ε” in the THz region. However, ε” has also been reported to increase in the THz region, indicating a shift in slow relaxation to higher frequencies, corresponding to an acceleration of slow relaxation.^19,27,67^ This so-called “negative hydration” is induced by the breaking of hydrogen bonds between water molecules.^18,68,69^ The acceleration phenomenon is discussed later. The fast relaxation mode of the isolated water molecules can be affected by solutes; the enhancement of this mode indicates a solute-induced isolation of water molecules from the hydrogen bond network, probably owing to the breaking of hydrogen bonds between water molecules.^68^ Because the stretching vibration mode had little effect on the spectrum in the frequency range analyzed in this study, changes owing to the solute were ignored. Because the real part of the dielectric constant, ε’, barely changes with the hydration effect and shows a larger standard deviation than the imaginary part (ε”), the change in these modes of water by osmolytes was evaluated by functional fitting with eq 2 ^49,51,60^ for ε” values of 0.3–2.5 THz.

2 $$\varepsilon \left(\omega \right)=c\left(\frac{\Delta \varepsilon_{\mathrm{slow}}}{1+i\omega \tau_{\mathrm{slow}}}+\frac{\Delta \varepsilon_{\mathrm{fast}}}{1+i\omega \tau_{\mathrm{fast}}}+\frac{A_{s}}{\omega_{s}^{2}-\omega^{2}+i\omega \gamma_{s}}\right)$$

where Δε_slow_ and Δε_fast_ are the intensities of slow and fast relaxation, respectively. The third term in the parentheses indicates the intermolecular stretching vibration mode between the water molecules. *A*_s_, ω_s_, and γ_s_ are the amplitude, resonant angular frequency, and damping constant, respectively. *c* is the volume fraction of water in the system, which was calculated for each solution using the solution density measured at room temperature (23.0 ± 1.0 °C) (see Supporting Information and Table S2). To fit the spectra, τ_slow_, τ_fast_, and the parameters of the stretching vibration were fixed to the corresponding values for pure water obtained by fitting the data for pure water at 20 °C. For fitting the pure water spectrum, τ_slow_, τ_fast_, ω_s_, and γ_s_ were taken from the literature^60^ (9.20 ps, 0.245 ps, 5.30 THz, and 32.5 THz). We confirmed that the obtained Δε_slow_ and Δε_fast_ (73.8 and 1.85), which dominantly affect the calculation of the hydration number, were in good agreement with previously reported results.^60^ By fitting the osmolyte solutions, *A*_s_ was fixed to be 35.0 THz^2^, which is in agreement with the values reported in previous studies.^27,70^

**Figure 3:**
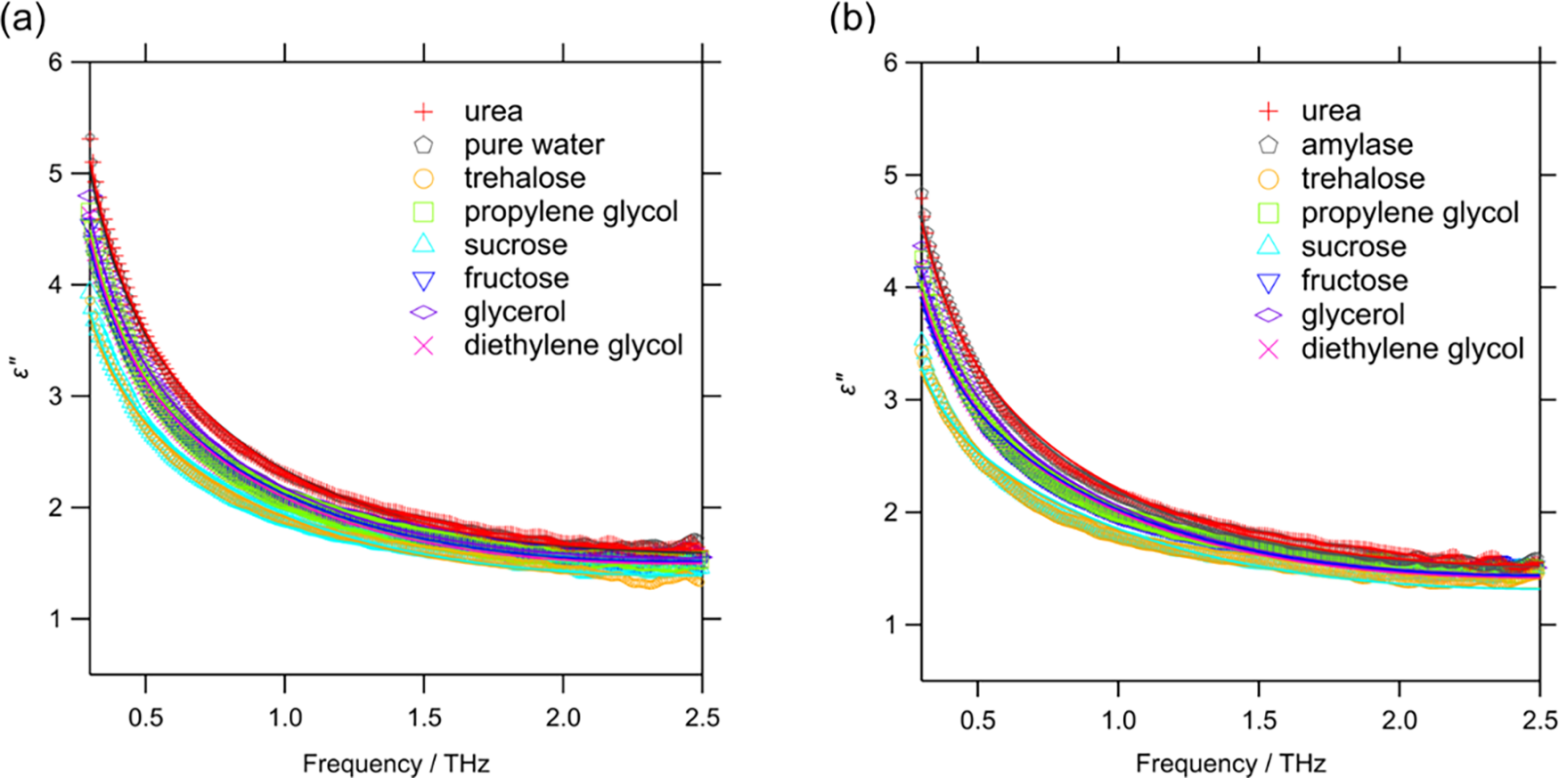
Imaginary part of the dielectric constant measured using THz-TDS for (a) each 0.75 mol/L osmolyte solution and (b) a solution of 10 wt% amylase with 0.75 mol/L of each osmolyte. The solid lines represent the fitting results by eq 2.

The intensities of the slow relaxation mode, Δε_slow_, in most of the osmolyte solutions were found to be lower than that of pure water. This reduction in intensity was due to a decrease in the amount of bulk water caused by the shift in the slow relaxation of hydration water to a much lower frequency. Thus, this decrease in Δε_slow_ indicates that water is bound to the osmolyte. Based on the decrease in Δε_slow_, the fraction *a*_hyd_ of the bound water molecules to all the water molecules in the system was determined using eq 3, taking into account Kirkwood’s correlation factor for slow and fast relaxations (2.9 and 1.0, respectively).^62^

3 $$a_{\mathrm{hyd}}=\left(\frac{\frac{\Delta \varepsilon_{\mathrm{slow}}^{\mathrm{water}}-\Delta \varepsilon_{\mathrm{slow}}^{\mathrm{solution}}}{2.9}-\frac{{\Delta \varepsilon }_{\mathrm{fast}}^{\mathrm{solution}}-{\Delta \varepsilon }_{\mathrm{fast}}^{\mathrm{water}}}{1}}{\frac{{\Delta \varepsilon }_{\mathrm{slow}}^{\mathrm{water}}}{2.9}+\frac{{\Delta \varepsilon }_{\mathrm{fast}}^{\mathrm{water}}}{1}}\right)$$

The superscripts “water” and “solution” refer to the results for pure water and osmolyte solution, respectively. The number of hydrated water molecules per osmolyte molecule, *n*_hyd_, was determined using eq 4

4 $$n_{\mathrm{hyd}}=\alpha \cdot a_{\mathrm{hyd}}$$

where α is the number of added water molecules per osmolyte molecule in the solution. Here, the slow and fast relaxations were assumed to be isolated from each other, and the bound hydration water was estimated from the total decrease in the slow relaxation of the bulk water and fast relaxation.^62^ (Fast relaxation often increased, resulting in a corresponding decrease in the hydration number.) The bound hydration water observed using THz-TDS is expected to include both strongly and weakly bound hydration water,^20^ which likely correspond to nonfreezing water and intermediate water, respectively.^71^ Inhibition of the collective rotational dynamics of water is likely caused by an increase in hydrogen bonding between water molecules induced by osmolytes.^68^ The dielectric constant of the urea solution in the THz region was higher than that of pure water. This increase was likely caused by a shift in the slow relaxation mode to higher frequencies than that of bulk water, indicating an acceleration of the collective rotational dynamics.^19,27,67^ Because comparing the hydration effects of all measured osmolytes in unified manner is important, eqs 3 and 4 were also applied to estimate the hydration effect of urea. In this case, the obtained Δε_slow_ is larger than that of pure water, and *n*_hyd_ becomes negative.^27^ In other words, *n*_hyd_ serves as an indicator of changes in water dynamics in terms of collective rotational dynamics. A highly positive *n*_hyd_ indicates a strong water-binding effect of the osmolytes, whereas a negative *n*_hyd_, by contrast, corresponds to an osmolyte-induced water-structure-breaking effect, resulting in accelerated water dynamics. The disruption of the hydrogen-bonded structure by urea^68,72^ can be assumed to accelerate the collective rotational dynamics. This structural breakdown of hydrogen bonds and accelerated water dynamics can only be investigated when observing hydration phenomena involving weakly hydrated water, which is enabled by THz spectroscopy. By contrast, recent molecular dynamics (MD) simulations have confirmed that many osmolytes that inhibit water rotational dynamics, such as sugars, increase hydrogen bonding between water molecules.^68^

Figure 4 shows a comparison of the *n*_hyd_ values for the osmolyte molecules measured using THz-TDS for the binary mixture of osmolytes and water with the normalized reaction rate constants (*k*/*k*_water_) for the respective osmolyte solutions. The results showed good correlation, with a correlation coefficient of *R* ^2^ = 0.91. Osmolytes with a large *n*_hyd_, which bind water effectively, decrease the reaction rate, whereas osmolytes with a negative *n*_hyd_, which increase water mobility, increase the reaction rate. This correlation strongly indicates that the change in water dynamics by osmolytes leads to a change in enzyme activity. Interestingly, urea accelerates the reaction rate despite the presence of a denaturant. These results are consistent with the proposed importance of water for enzyme activity.^15,22^ Enzymes in organic solvents are more active than those in water, despite being denaturant,^73^ which also confirms that well-hydrated enzymes become less active.

**Figure 4:**
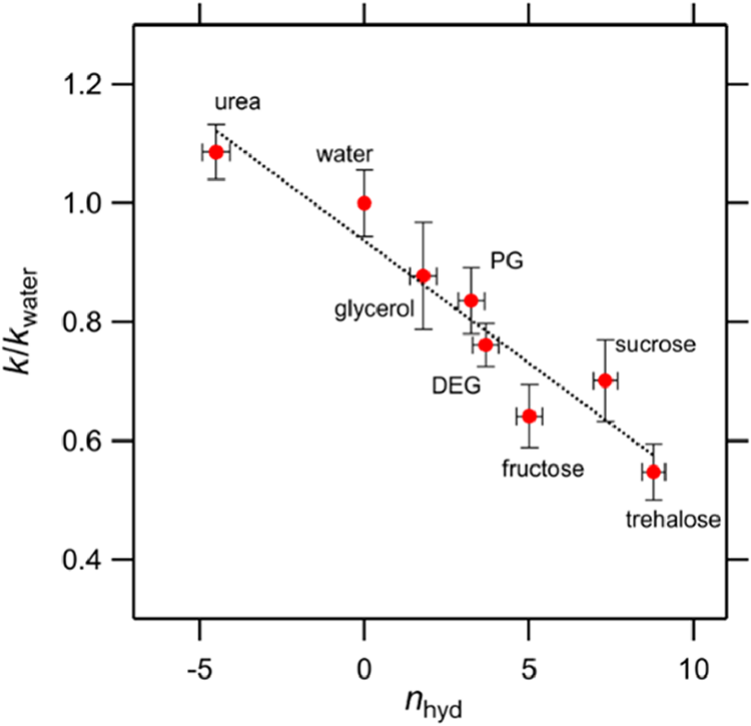
Normalized reaction rate constant (*k*/*k*_water_) of amylase with/without respective osmolytes compared with the mobility change in the collective rotational dynamics of water (*n*_hyd_), measured using THz-TDS. A positive *n*_hyd_ indicates inhibited mobility, whereas a negative *n*_hyd_ indicates that water mobility is enhanced by the osmolyte. If *n*_hyd_ is positive, its value is equal to the number of water molecules bound to each osmolyte molecule. The error bars for *n*_hyd_ indicate the deviation when *A*_s_ was changed from 33.0 THz^2^ to 37.0 THz^2^. The dotted line represents the linear fitting (*R* ^2^ = 0.91).

THz-TDS was also performed on the amylase/osmolyte/water ternary system (Figure 3(b)). Because water is affected by amylase and osmolytes in ternary systems, the hydration effects were evaluated based on the fraction of hydrated water in the entire system, *a*_hyd_, rather than the amount of hydration water per molecule. In Figure 5, the obtained *a*_hyd_ values for the solutions of 10 wt% amylase and 0.75 M osmolyte are plotted against the normalized reaction rate constant (*k*/*k*_water_). The results correlate well (*R* ^2^ = 0.89), similar to the correlation between *n*_hyd_ and *k*/*k*_water_. This indicates that the effects of the osmolytes dominated the dynamics of water, even in ternary solutions. This correlation strongly indicates that the change in water dynamics by osmolytes leads to a change in enzyme activity.

**Figure 5:**
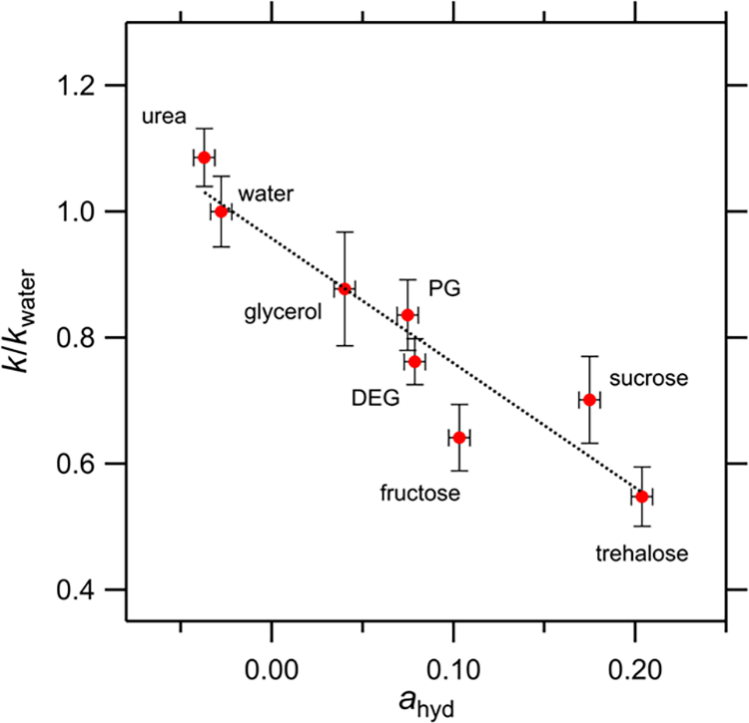
Normalized reaction rate constant (*k*/*k*_water_) of amylase with/without respective osmolytes compared with the degree of mobility change in the collective rotational dynamics of water (*a*_hyd_) in solutions containing 10 wt% amylase and 0.75 M osmolyte, measured using THz-TDS. Positive *a*_hyd_ values indicate bound water dynamics, while negative values indicate acceleration of the relaxation dynamics. The error bars for *a*_hyd_ indicate the deviation when the fixed *A*_s_ value was changed from 33.0 THz^2^ to 37.0 THz^2^. The dotted line represents the linear fitting result (*R* ^2^ = 0.89).

These results demonstrate that the mobility of water in terms of collective rotational dynamics on picosecond time scales is an important factor in determining the ease of enzymatic reactions. Osmolytes are proposed to indirectly affect enzymes through the dynamical change of water molecules. The finding that the mobility of the surrounding water correlates with enzyme activity is unsurprising, as previous MD simulation studies^15^ have revealed that osmolytes alter the dynamics of water molecules and proteins that facilitate the binding of DNA to proteins. It is also important that the correlation is viewed in a unified manner, beyond the molecular species of the various osmolytes. The indirect effect of osmolytes via water is consistent with an MD simulation study^74^ that revealed that osmolytes such as glycine, betaine, and TMAO do not interact directly with proteins, but slow the rotation dynamics of water molecules on a picosecond time scale.

Although the mechanism of the correlation between the dynamics of water molecules affecting enzyme activity is unclear, the likely scenario is that the enzymes’ tertiary and/or secondary structures and their dynamics are changed owing to the change in the dynamics of the surrounding water,^75^ which contributes to enzyme activity. On the other hand, past studies of circular dichroism spectroscopy have demonstrated that osmolytes do not directly alter enzyme tertiary structure.^76,77^ Thus, the change in enzyme activity would likely stem from alterations in protein dynamics, such as changes in flexibility, by the change in water dynamics. Nuclear magnetic resonance (NMR) studies have revealed that the dynamics of proteins are coupled to those of water molecules.^78^ Furthermore, the flexibility of the protein may also contribute to substrate binding.^79^ A MD simulation study^80^ also found that lipase activity increases as the water activity in the system increases. In this case, the solute-accessible surface area (SASA) of the lipase increased with increasing water activity, leading to a change in the local conformation of the active site pocket of the enzyme. The water dynamics for ligand binding of proteins, such as myoglobin,^11,12^ is important, and osmolytes have been reported to indirectly alter the structure of myoglobin through water around proteins and heme pockets.^36^ These studies imply that osmolytes change the dynamics of water molecules around the active site, thereby altering its local structure and dynamics of the active site. It is possible that a change in the local structure of the active site facilitates binding of the substrate to the site, leading to higher enzyme activity. Urea may increase the mobility of water molecules around the enzyme, imparting flexibility, such as the dynamics of the active site’s side chains, which may increase enzyme activity. At present, it is not known at which stage of the amylase enzyme reaction, that is at formation of enzyme-sugar intermediate state or at the hydrolytic cleavage of amylose in the active site,^55,56^ water is most involved. It is conceivable that added urea makes water molecules more mobile, making it easier for water molecules to attack enzyme-sugar intermediate state for hydrolysis. It is also possible that osmolytes directly interact with enzyme’s active sites or substrates,^76^ and that osmolytes affect the binding affinity of molecules to the active site of an enzyme.^81^ However, the results of the present study indicate that various osmolytes, such as polyols and sugars, follow a single trend, suggesting that indirect effect of osmolyte through change in water state is dominant for the activity change.

In addition to the effects of water dynamics on the folded structures and the dynamics of enzymes, other mechanisms should also be considered. Our finding that urea enhanced enzyme activity—despite being a denaturant—implies that hydrogen bonds between water molecules around the active site may be broken by urea,^68^ which facilitates substrate access to the active site. Conversely, other osmolytes can structure water better than bulk water^68^ and prevent the substrates from approaching the active site. Not only the dynamics and structure of enzymes, but also those of substrates, can be related to enzymatic reactions. Because hydrolysis is the reaction catalyzed by amylase, the change in water dynamics owing to the osmolytes likely affects the hydrolysis reaction. More detailed studies on the mechanisms underlying the link between water dynamics and enzyme activity are required in the future.

## Conclusion

In this study, we systematically evaluated the effect of osmolytes on enzyme activity from the perspective of water dynamics. Previous studies on osmolytes have primarily focused on protein structural stabilization and denaturation inhibition. However, their effects on protein function, i.e., enzyme reaction rates, and the role of hydration state are not fully understood. We investigated the hydration state of seven osmolytes to evaluate how the associated water dynamics affects the reaction rate of amylose degradation by α-amylase. Using THz spectroscopy to measure the collective rotational dynamics of water clusters, we found that the changes in water dynamics induced by each osmolyte correlated with the changes in enzyme activity. Adding osmolytes such as urea increased the mobility of water molecules and accelerated enzyme reactions, whereas osmolytes such as sugars, which inhibit the mobility of water molecules, slowed enzyme reactions. This correlation was consistently observed not only in the binary systems of osmolytes and water but also in the ternary systems of enzymes, osmolytes, and water. This correlation strongly indicates that osmolytes affect water dynamics, and that changes in water molecular dynamics influence enzyme activity. In other words, osmolytes indirectly affect the enzyme activity via water dynamics. This is similar to the mechanism through which osmolytes indirectly affect protein stability through water^27^ as well as the Hofmeister effect, which explains the protein stabilizing/destabilizing effect of salt.^38,39^ The present study experimentally demonstrates that enzymatic reactions are sensitive to the picosecond-scale motion of water molecules. Interestingly, urea promotes enzyme activity despite being a denaturant, which can be explained by the water dynamics. These findings deepen our understanding of the molecular mechanisms underlying these enzymatic reactions and provide new guidelines for the utilization of osmolytes in food science, biotechnology, and drug discovery.

## Supplementary Material



## Notes

Visible light absorption spectrum of the amylose/iodine solution, Normalized reaction rate constants of all samples, Change in reaction rate constants, Table of averaged normalized reaction rate constants of all samples, Table of density of each osmolyte solution (PDF)

## Abbreviations

THz-TDS: terahertz time domain spectroscopy
PG: propylene glycol
DEG: diethylene glycol
MD: molecular dynamics
