# Supplementary material for: Changes in Water Dynamics by Osmolytes Regulate Enzyme Activity
Source: J Phys Chem B. 2026 Jan 6;130(3):992–1000. doi: 10.1021/acs.jpcb.5c07605 (PMC12833849; doi:10.1021/acs.jpcb.5c07605)
Supplement: Supplementary file 1 [file jp5c07605_si_001.pdf]

Supporting Information

Changes in Water Dynamics by Osmolytes Regulate Enzyme Activity

*Sachika Furukawa and Mafumi Hishida**

Department of Chemistry, Faculty of Science, Tokyo University of Science, 1-3 Kagurazaka, Shinjuku, Tokyo 162-8601, Japan

* hishida@rs.tus.ac.jp

The absorption spectrum of the amylose/iodine solution is shown in Figure S1. Because the absorption peak appears at 615 nm, time-dependent measurements of the UV–visible absorbance for the iodine–starch reaction were performed at 615 nm.

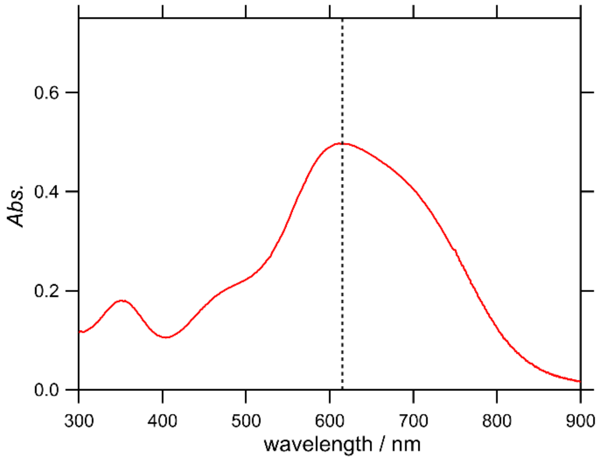

Figure S1. Absorption spectrum of amylose/iodine solution. The dotted line indicates 615 nm.

The normalized reaction rate constant of the iodine–starch reaction by amylase after adding each osmolyte is shown in Figure S2. As shown in the main text, the rate constants for the reactions with osmolytes were normalized by the reaction rate constants without osmolytes when an amylose solution taken from the same sample bottle was used. The average value was used as the reaction rate constant for each osmolyte, and the standard deviation was used as the error (Table S1).

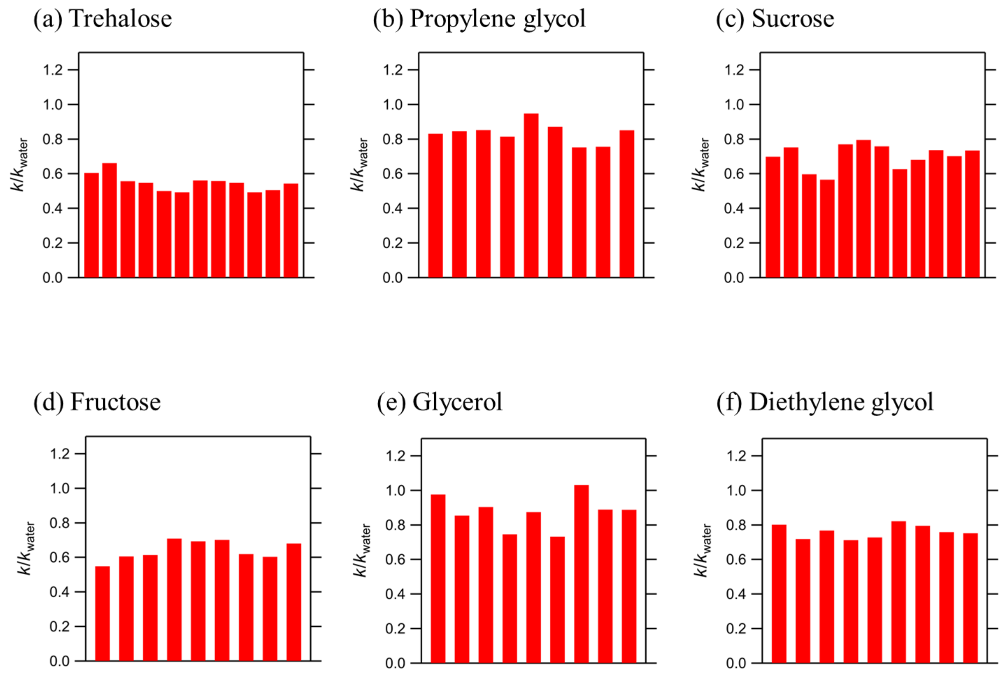

Figure S2. Normalized reaction rate constants of the iodine–starch reaction by amylase upon addition of 0.75 mol/L of each osmolyte. (a) Trehalose, (b) propylene glycol, (c) sucrose, (d) fructose, (e) glycerol, and (f) diethylene glycol.

Table S1. Normalized reaction rate constants of the iodine–starch reaction of amylase in each 0.75 mol/L osmolyte solution and their standard deviations.

Osmolyte	k/k_{water}	Standard Deviation
Urea	1.10	0.05
Trehalose	0.55	0.05
Propylene glycol	0.84	0.06
Sucrose	0.70	0.07
Fructose	0.64	0.05
Glycerol	0.88	0.09
Diethylene glycol	0.76	0.04

The reaction rate constants up to three days after preparing the amylose solution without osmolytes are shown in Figure S3. The reaction rate constant, k, decreased with the number of days after preparation. Therefore, all experiments were conducted at two days after preparing the amylose solutions.

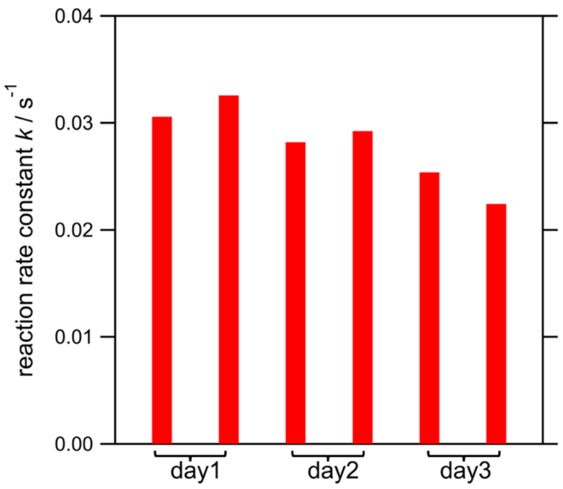

Figure S3. Reaction rate constants for the same amylose solution without osmolyte up to 3 d after sample preparation.

The volume fraction of water in system c in Equation (2) for each solution was calculated from the solution density measured at room temperature (23.0 ±1.0 °C) using DMA 35 (Anton Paar GmbH). The measured densities and c values are summarized in Table S2.

Table S2. Solution density of each osmolyte solution and calculated volume fraction, c, of water in the system

Osmolyte	Density / g cm^{-3}	c
None	0.9968	1
Urea	1.0082	0.9689
Trehalose	1.0929	0.8463
Propylene glycol	1.0007	0.9448
Sucrose	1.0947	0.8251
Fructose	1.0473	0.9269
Glycerol	1.0127	0.9459
Diethylene glycol	1.0079	0.9298
